# Loss of function mutation of *Eftud2*, the gene responsible for mandibulofacial dysostosis with microcephaly (MFDM), leads to pre-implantation arrest in mouse

**DOI:** 10.1371/journal.pone.0219280

**Published:** 2019-07-05

**Authors:** Marie-Claude Beauchamp, Anissa Djedid, Kevin Daupin, Kayla Clokie, Shruti Kumar, Jacek Majewski, Loydie Anne Jerome-Majewska

**Affiliations:** 1 Department of Pediatrics, McGill University, Montreal, QC, Canada; 2 McGill University Health Centre at Glen Site, Montreal, QC, Canada; 3 Department of Human Genetics, McGill University, Montreal, QC, Canada; 4 Department of Anatomy and Cell Biology, McGill University, Strathcona Anatomy and Dentistry Building, Montreal, Quebec; Medical College of Wisconsin, UNITED STATES

## Abstract

Mutations in *EFTUD2* are responsible for the autosomal dominant syndrome named MFDM (mandibulofacial dysostosis with microcephaly). However, it is not clear how reduced levels of *EFTUD2* cause abnormalities associated with this syndrome. To determine if the mouse can serve as a model for uncovering the etiology of abnormalities found in MFDM patients, we used *in situ* hybridization to characterize expression of *Eftud2* during mouse development, and used CRISPR/Cas9 to generate a mutant mouse line with deletion of exon 2 of the mouse gene. We found that *Eftud2* was expressed throughout embryonic development, though its expression was enriched in the developing head and craniofacial regions. Additionally, *Eftud2* heterozygous mutant embryos had reduced EFTUD2 mRNA and protein levels. Moreover, *Eftud2* heterozygous embryos were born at the expected Mendelian frequency, and were viable and fertile despite being developmentally delayed. In contrast, *Eftud2* homozygous mutant embryos were not found post-implantation but were present at the expected Mendelian frequency at embryonic day (E) 3.5. Furthermore, only wild-type and heterozygous E3.5 embryos survived *ex vivo* culture. Our data indicate that *Eftud2* expression is enriched in the precusor of structures affected in MFDM patients and show that heterozygous mice carrying deletion of exon 2 do not model MFDM. In addition, we uncovered a requirement for normal levels of *Eftud2* for survival of pre-implantation zygotes.

## Introduction

Heterozygous mutations in *EFTUD2* (elongation factor tu GTP binding domain containing 2, also called Snu114) are responsible for mandibulofacial dysostosis with microcephaly (MFDM) [1–3]. A variety of clinical features are associated with MFDM [4], though the most common features are: micrognathia, small or dysplastic pinna(e), malar hypoplasia, hearing loss and auditory atresia/stenosis. Importantly, all patients exhibit developmental delay, 88% of them have microcephaly, and all carry a mutation in *EFTUD2* [4, 5]. Since 2012 when Lines *et al*. [6] reported that mutations in *EFTUD2* are responsible for MFDM, 86 distinct mutations have been described in this gene. To date, 7 large deletions, 16 frameshift, 13 nonsense, 35 splice site, one small deletion/duplication, and 14 missense [3–5, 7, 8] have been reported in *EFTUD2*. Furthermore, though 75% of these mutations are *de novo* and 6% are due to germline mosaicism, 19% are inherited from an affected parent [4, 5]. In addition, though mutations in *EFTUD2* are distributed along the length of the gene, no phenotype-genotype correlation has been found [4].

*EFTUD2* encodes a GTPase that is highly conserved in eukaryotes and is a core module of the U5 small nuclear ribonucleoprotein (snRNP) component of the spliceosome. Although it is clear that both the GDP/GTP state and phosphorylation status of EFTUD2 contribute to splicing and disassembly of the spliceosome [9, 10], the factors which regulate EFTUD2 status remains to be identified.

Mutations in zebrafish eftud2 were generated using N-ethyl-N-nitrosurea (ENU) mutagenesis and TALENs [11, 12]. In both studies, increased apoptosis was found in the head region, mostly the brain and retina, and the spinal cord. In addition, Lei *et al*. showed that *eftud2* was required for survival of neural progenitors in a p53-dependent manner [11]. Interestingly, in both zebrafish models, heterozygous animals did not show any phenotypic or molecular abnormalities, while a microcephalic phenotype reminescent of that found in MFDM patients was observed in homozygous mutant animals. In the present study, we characterize expression of *Eftud2* during mouse embryogenesis, and report generation of the first mouse model carrying *Eftud2* mutation. We show that heterozygous mice on a mixed CD1;FvB or inbred C57BL/6 genetic background does not model MFDM, and that *Eftud2* is essential in pre-implantation zygotes.

## Results

### *Eftud2* shows tissue-specific expression during early development

**Fig 1:**
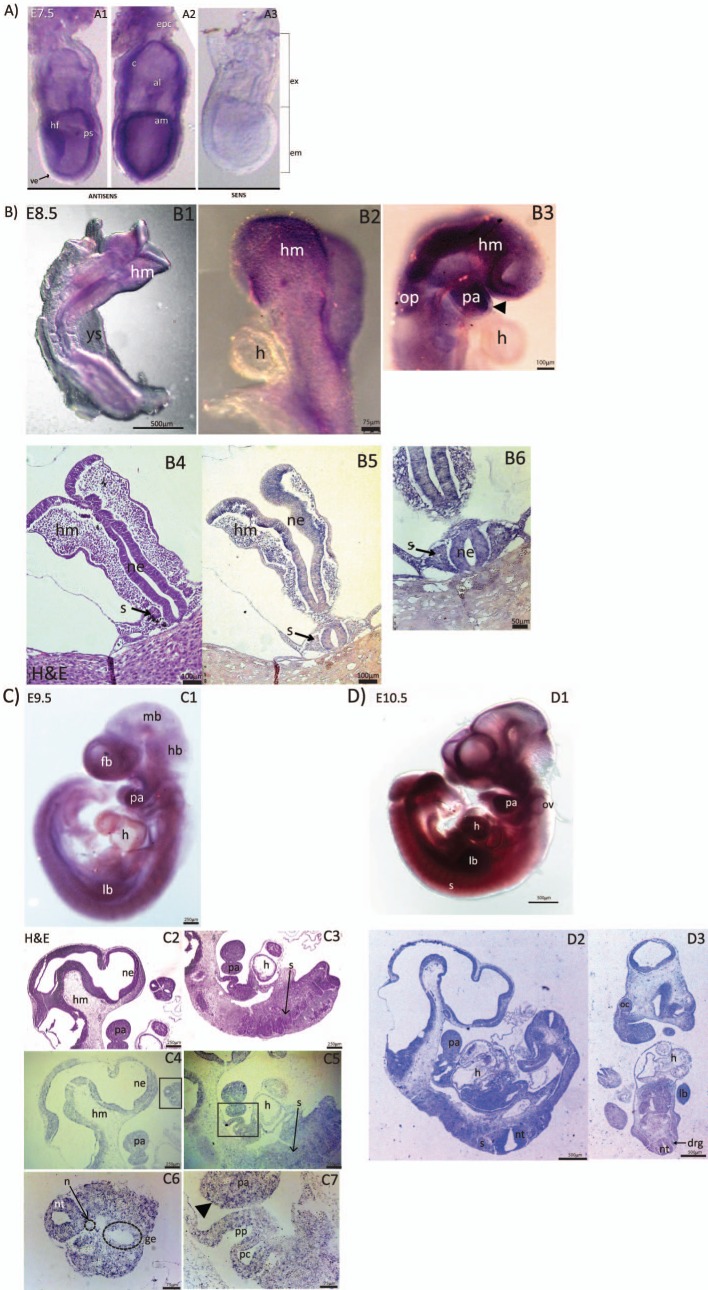
Representative images from *in situ* hybridation (ISH) showing *Eftud2* expression in wild-type CD1 embryos at various developmental stages. **A)** Wholemount ISH on E7.5 embryos using antisense (A1-A2) and sense probes (A3). *Eftud2* is expressed in both embryonic (em) and extra-embryonic (ex) region. *Eftud2* is found in headfold (hf), primitive streak (ps), amnion (am), allantois (al), chorion (c) and ectoplacental cone (epc), but not in visceral endoderm (ve). **B)** Wholemount and section ISH on E8.5 embryos. *Eftud2* was expressed in head mesenchyme (hm), pharyngeal arches (pa) and otic placode (op) (B1-B3). No expression was found in the heart (h) (B2-B3). Headarrow points to the ectoderm of pa (B3). H&E staining of region examined in B5 (B4). Sections ISH reveals expression in head mesenchyme (hm), somites (s) and neural epithelium (ne) (B5-B6). **C)** Wholemount (C1) and ISH on sagittal sections (C4-C7) of E9.5 embryos. Expression was found in the forebrain (fb), midbrain (mb), hindbrain (hb), pharyngeal arches (pa) and limb buds (lb) while reduced expression was found in the heart (h) (C1). H&E staining of regions stained is shown (C2-C3). ISH on sagittal sections shows enriched expression in neural epithelium (ne) and pharyngeal arches (pa) (C4). Reduced expression was found in the heart (h), and the somite (s) (C5). Higher magnification image of the boxed region in C4 indicate that *Eftud2* was also expressed in the neural tube (nt) and gut epithelium (ge) but not in the notochord (n) (C6). Higher magnification image of the boxed region in C5 shows expression in pharyngeal pouches (pp) and paryngeal clefts (pc) and in non-neural ectoderm (arrowhead) (C7). **D)** Wholemount (D1), ISH on sagittal (D2) and frontal (D3) sections of E10.5 embryos. At this stage, expression was ubiquitous and included otic vesicles (ov), dorsal root ganglia (drg) and optic cup inner layer (oc).

To determine when *Eftud2* is first expressed in organs affected in MFDM patients, we performed *in situ* hybridization and characterized expression of this gene in wild-type embryos from E7.5 to E10.5 (Figs 1 and S1). At E7.5, *Eftud2* was not expressed in visceral endoderm, but was detected in headfolds, the primitive streak, the amnion, the ectoplacental cone, the chorion, and the allantois (Fig 1A1 and 1A2). A day later, at E8.5, *Eftud2* was not expressed in the heart (Fig 1B2 and 1B3), and reduced expression was found in non-neural ectoderm overlying the first pharyngeal arch (arrowhead) and the developing forebrain, when compared to the associated mesenchyme (Fig 1B3). In contrast, expression of *Eftud2* was enriched in the developing head and brain, and the pharyngeal arches and otic placode (Fig 1B1–1B3). *In situ* hybridization on sections of E8.5 embryos also revealed expression of *Eftud2* in the neural epithelium and somites (Fig 1B5 and 1B6).

At E9.5, expression of *Eftud2* was reduced in the notochord and cardiac region when compared to neighboring tissues (Fig 1C5 and 1C6). However, it was uniformly expressed in the developing brain, and the pharyngeal apparatus which will form the face (Fig 1C4–1C7). At this stage, *Eftud2* was also expressed in non-neural ectoderm (arrowhead) (Fig 1C7). Finally, at E10.5, expression of *Eftud2* was widespread and evenly detected in the developing head and face: forebrain, midbrain and hindbrain, otic vesicles, pharyngeal arches and the optic cup (Fig 1D1); in the neural tube, dorsal root ganglia, somites, heart, and limb buds (Fig 1D2 and 1D3) [13]. Thus, *Eftud2* was expressed in precursors of tissues affected in MFDM patients prior to and after the onset of organogenesis.

### Deletion of exon 2 of mouse *Eftud2* results in reduced mRNA and protein levels

**Fig 2:**
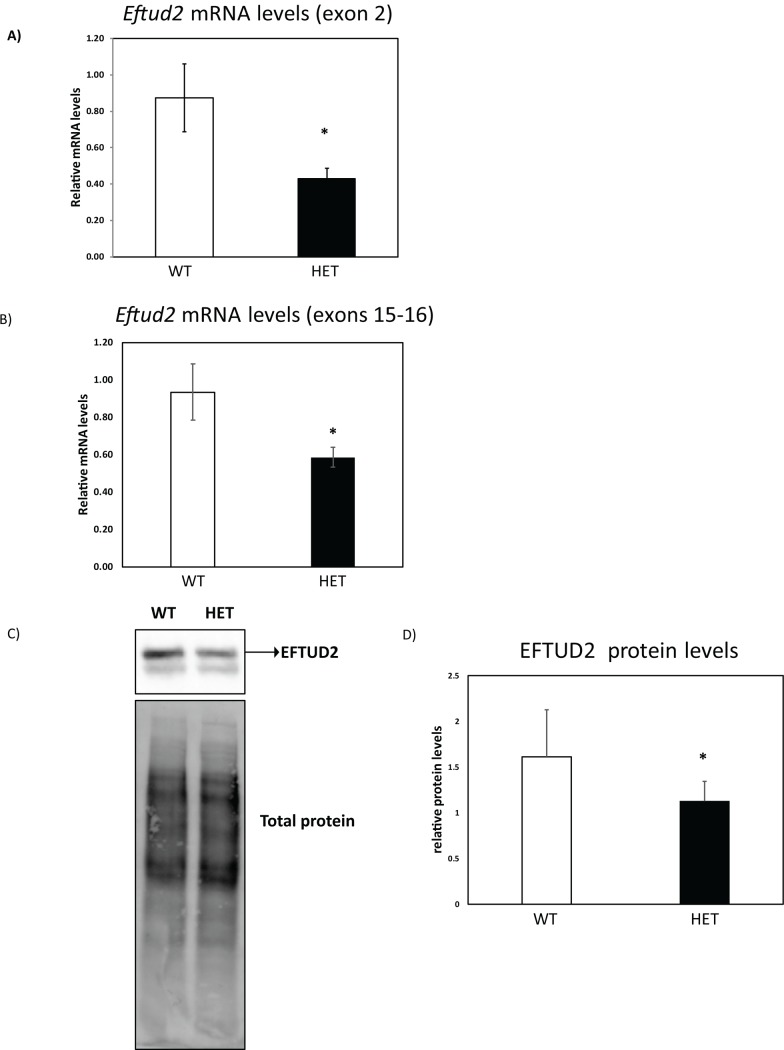
Reduced Eftud2 mRNA and protein levels in heterozygous mice. *Eftud2* mRNA levels was evaluated using RT-qPCR in E9.5 embryos on the mixed genetic background with primers flanking **A)** exon 2 or **B)** exons 15–16. WT = 3, HET = 3 (see Mat&Methods section for samples description). **C)** Total proteins from E11.5 embryos on the mixed genetic background were subjected to Western blot as described in the Mat&Methods section. One representative blot of 3 independent experiments is shown and **D)** quantification of EFTUD2 relative to total protein level. WT = 5, HET = 15. Results represent average ± SD. *P<0.05 by t-test.

To determine if heterozygous mutation of *Eftud2* in mouse models MFDM, we used CRISPR/Cas9 to delete exon 2 of *Eftud2* on two genetic backgrounds: a mixed (CD1;FvB) and an inbred (C57BL/6). Deletion of exon 2, the first coding exon of the gene, is predicted to result in splicing into exon 3 and the generation of an alternatively spliced *Eftud2* isoform that has been annotated in human, but not in mouse. In addition, this alternatively spliced isoform is predicted to encode for a truncated protein that is missing the first 35-amino acids at the N-terminal portion of EFTUD2. To examine the consequence of the engineered deletion of exon 2, we compared levels of *Eftud2* mRNA and EFTUD2 protein in wild-type and *Eftud2* heterozygous (*Eftud2*^*+/-*^) embryos on the mixed genetic background. As shown in Fig 2, RT-qPCR with primers flanking the deleted exon 2 (Fig 2A) or exons 15–16 (outside of the deletion) (Fig 2B) revealed a significant reduction in *Eftud2* mRNA levels in E9.5 *Eftud2*^*+/-*^ embryos, compared to wild-type (t-test, P<0.05). Furthermore, western blot analysis with an antibody raised against the carboxyl-terminal region of EFTUD2 showed a statistically significant 30% reduction in EFTUD2 protein levels in E11.5 *Eftud2*^*+/-*^ embryos when compared to wild- type (Fig 2C and 2D) (t-test, P<0.05). Hence, we conclude that levels of EFTUD2 mRNA and protein are reduced in *Eftud2*^*+/-*^ mouse embryos with deletion of exon 2.

### Reduced levels of EFTUD2 do not perturb expression of genes in the P53 pathway or in non-sense mediated decay (NMD)

Zebrafish eftud2 mutants have upregulation of genes in the P53 pathway [11]. Therefore, we assessed *Eftud2*^*+/-*^ embryos on the mixed genetic background for pertubation in this pathway. As shown in S2A Fig, the mRNA level of genes in the P53 pathway which were upregulated in zebrafish morphants: *caspase 8*, *Bbc3* and *Trp53* was comparable between wild-type and *Eftud2*^*+/-*^ embryos, at E9.5. Moreover, since it was postulated that reduced levels of EFTUD2 overwhelms the nonsense mediated pathway, we evaluated expression of a key component of NMD, *Upf1*. Although a slight reduction in *Upf1* mRNA level was observed (S2B Fig), this decrease did not reach statistical significance. Thus, our data demonstrates that a 30% reduction of EFTUD2 protein was not sufficient to activate the p53 pathway or disrupt NMD in mouse.

### The transcriptional landscape of *Eftud2*^*+/-*^ embryos is not disrupted

To explore if reduced EFTUD2 protein level was associated with any transcriptional changes, RNAseq was performed to examine transcriptomes of E9.5 wild-type and *Eftud2*^*+/-*^ embryos on the mixed genetic background. A Principal Component Analysis (PCA) on all genes and hierarchical clustering on the 1000 most variable genes revealed that heterozygous and wild-type embryos did not cluster based on their genotype, but according to their litter (S3 and S4 Figs), suggesting that maternal environment differences are more important than any differences caused by reduced levels of EFTUD2. Furthermore, differential expression analysis revealed that *Eftud2* heterozygous embryos did not have any genes that were down-regulated, and that only four genes showed up-regulated expression (see S1 Table).

To determine if splicing was disrupted in *Eftud2*^*+/-*^ embryos, junction usage was analyzed. Using a p-value cutoff of 0.05 and a junction usage difference greater than or equal to 0.20 between heterozygous and wild-type embryos, only nine genes were identified as differentially spliced (supplemental data). In addition, these splicing changes were not found in genes upregulated at the transcript level. In fact, the junction with the greatest usage difference was in *Eftud2*, and corresponds to the deletion of exon 2 introduced in the DNA of mutant embryos. As depicted in S5 Fig, exon 2 of *Eftud2* gene was absent in 50% of all *Eftud2* transcripts of heterozygous mutant embryos, an expected consequence of the deletion, while always included in transcripts from wild-type embryos. Furthemore, when the analysis was repeated with a False Discovery Rate (FDR) equal to 0.05, no statistically significant differences was found in junction usage between wild-type and heterozygous embryos.

To rule out the possibility of increase usage of illegitimate splice junctions, the percentage of unannotated junctions found in wild-type and *Eftud2*^*+/-*^ mutant embryos were compared. However, this analysis also revaled no statistically significant difference, even at the global level (S2 and S3 Tables). Thus, our transcriptional analysis showed that deletion of *Eftud2* exon 2 has a minor effect on transcription, and no statistically significant impact on splicing in heterozygous embryos.

### *Eftud2*^*+/-*^ embryos are developmentally delayed between E8.5 and E9.5

**Fig 3:**
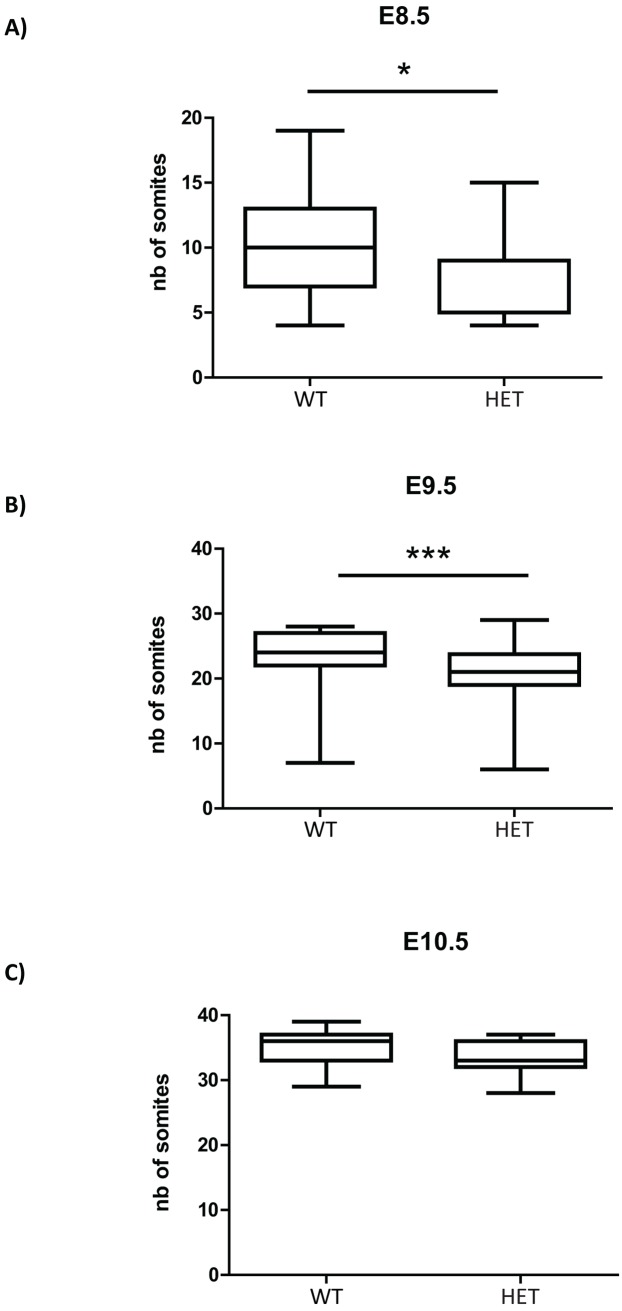
*Eftud2* heterozygous embryos on the mixed CD1:FVB genetic background have reduced number of somites at E8.5 and E9.5. Somites counted at **A)** E8.5 WT = 11; HET = 27 **B)** E9.5, WT = 32; HET = 72 **C)** E10.5, WT = 15; HET = 40. *P<0.05, ***P<0.001 by t-test.

We next examined *Eftud2*^*+/-*^ embryos for morphological abnormalities in structures affected in MFDM patients. At E8.5 and E9.5, *Eftud2* heterozygous mutant embryos on the mixed CD1;FvB genetic background had 2–3 pairs of somites less than their wild-type littermates (Fig 3A and 3B; P<0.05, t-test), indicating these embryos were developmentally delayed. However, this reduction was no longer statistically significant at E10.5 (Fig 3C). In fact, before birth, no significant difference was found in weights of E18.5 wild-type and heterozygous embryos or their placentas, as shown in S6 Fig. Altogether these findings indicate that heterozygous embryos are delayed when compared to their wild-type littermates before the onset of organogenesis, but catch up by birth.

### *Eftud2*^*+/-*^ mice do not model MFDM and are viable and fertile

To evaluate if *Eftud2* heterozygous mice were born at the expected Mendelian frequency and survived the perinatal period, litters were monitored from postnatal day (P)0 to P21 on both genetic background. On the mixed CD1;FvB genetic background, *Eftud2* heterozygous mice were found at the expected Mendelian frequency (Table 1). In addition, gross analysis and skeletal preparation of wild-type and heterozygous pups revealed no morphological abnormalities in these mice (data not shown). Thus, the statistically significant 30% reduction in EFTUD2 mRNA and protein levels did not impact embryonic development or viability of *Eftud2* heterozygous mice on this genetic background.

In contrast, on the inbred C57BL/6 genetic background, Chi-square analysis revealed that the number of *Eftud2* heterozygous pups present at weaning were significantly less than expected (Table 1) (chi-square test, p<0.05). Therefore, we collected embryos from mating of heterozygous male and females and from mating of heterozygous male with wild-type females to determine if *Eftud2* heterozygous embryos die before birth. Our analysis revealed that both mating schemes generated heterozygous embryos at the expected Mendelian frequency (Tables 2 and 3, respectively). Furthermore, *Eftud2* heterozygous embryos and pups were indistinguishable from their wild-type littermates before birth and at P0. Thus, heterozygous pups on the inbred C57BL/6 genetic background did not model abnormalities associated with MFDM. We presume that *Eftud2* heterozygous pups on this inbred genetic background die shortly after birth due to unknown causes and are cannibalized by their mother.

**Table 1:** Genotype of mice weaned after breeding of *Eftud2*^*+/-*^ and wild-type mice.

	WT	HET	n.t.	#litters
CD1;FvB	312 (1)	285	17 (17)	40
C57BL/6	62	36 (1)**	9 (9)	16

**λ^2^ test P<0.01

n.t. not typed, dead animals between birth and weaning are in parenthesis

**Table 2:** Genotype of embryos after mating of *Eftud2*^*+/-*^ and wild-type mice on the C57BL/6 genetic background.

	WT	HET	n.t.	#litters
E15.5	3	5	1 (1)	1
E18.5	12	8	0	3

Resorptions are in parenthesis.

### *Eftud2*^*-/-*^ embryos are not found post-implantation

To determine if mice with homozygous mutation in *Eftud2* models MFDM, *Eftud2*^*+/-*^ male and females were mated. As shown in Tables 3 and 4, on both the mixed CD1;FvB and the inbred C57BL/6 genetic backgrounds, *Eftud2* homozygous mutants embryos were only recovered at E3.5 (Chi square test, P<0.001, at all other stages). Interestingly, excluding E3.5, the expected Mendelian ratio of 1:2 of wild-type and heterozygous embryos was observed (Chi square test, P = 0.81 in total embryos), on both the mixed and inbred genetic backgrounds, consistent with our conclusion that *Eftud2* heterozygous embryos do not arrest during embryogenesis. However, these data demonstrate that *Eftud2*^*-/-*^ embryos do not survive post-implantation.

**Table 3:** Genotypes of embryos collected from matings of *Eftud2*^*+/-*^ with *Eftud2*^*+/-*^ mice on the C57BL/6 genetic background.

stage	(+/+)	(+/-)	(-/-)	n.t.	TOTAL	litters
E3.5	3	9	2	0	14	3
E9.5	1	11	0	1 (1)	12	2
E11.5	1	3	0	1 (1)	4	1
E16.5	2	2	0	1 (1)	4	1
E17.5	1	7	0	2 (2)	8	2
**TOTAL**	**8**	**32**	**2**	**5 (5)**	42*	**9**

*λ^2^ test P<0.001 in total. Resorptions are in parenthesis.

n.t. not typed

**Table 4:** Genotype of embryos collected from matings of *Eftud2*^*+/-*^ with *Eftud2*^*+/-*^ mice on the mixed CD1;FvB genetic background.

stage	(+/+)	(+/-)	(-/-)	n.t.	TOTAL	# litters
E3.5	37	63	42	17	142	14
E7.5	10	19	0	7 (6)	29	4
E8.5	9	31	0	19 (15)	40	4
E9.5	39	72	0	23 (21)	111	10
E10.5	17	43	0	16 (16)	60	7
E11.5	5	26	0	9 (9)	31	3
E14.5	27	41	0	15 (14)	68	7
E16.5	14	25	0	11 (11)	39	5
E17.5	15	17	0	6 (6)	32	3
E18.5	18 (2)	34 (2)	0	18 (18)	56	6
**TOTAL**	**191 (2)**	**371 (2)**	**42**	141 (116)	608*	**63**

*λ^2^ test P<0.001 at all stages, except at E3.5, P = 0.3405.

Resorptions are in parenthesis. n.t. not typed

### *Eftud2* homozygous mutant do not survive *ex vivo*

We cultured embryos from *inter se* matings of wild-type or *Eftud2* heterozygous mice on the mixed genetic background *ex vivo* to assess their developmental potentials. E3.5 embryos were cultured for 5 days, and scored each day using the following criteria: compacted morula–embryos with no discernible cell-boundaries and no blastocyst cavity; blastocyst–embryos with a blastocyst cavity which occupies less than 50% of the embryo volume; expanded blastocysts–embryos with a blatocyst cavity which occupy more than 50% of the embryo volume; hatched blastocysts–embryos without a zona pellucida; inner cell mass formation and trophectoderm outgrowth–presence of a discernible inner cell mass (ICM) and outgrowth of the trophectoderm; dead–degenerated embryos with cellular debris (S7 Fig).

As expected, 100% of embryos from wild-type matings survived the 5 days of culture (n = 25, S4 Table). At the end of the 5-day culture, 88% of these embryos were hatched from the zona pellucida (n = 22/25), and 72% had formed cultures with ICM and trophectoderm outgrowth (n = 18/25). The remaining embryos were expanded blastocysts that had not hatched from their zona pellucida.

In contrast, although 75% of E3.5 embryos from mating of *Eftud2*^*+/-*^ males and females were alive and healthy (n = 33/44), 25% failed to survive the 5-days *ex vivo* culture (n = 11/44) Table 5). Of surviving embryos, 91% hatched from their zona pellucida and 88% formed outgrowths containing ICM and trophectoderm, similar to wild-type. Most embryos which died formed a blastocoel cavity (n = 8/11), though a number arrested as morulas (uncompacted morulas n = 1/11, compacted, n = 2/11). When a subset of blastocysts were genotyped after the 5 days of culture, we found that wild-type (n = 4) and heterozygous (n = 9) embryos gave rise to ICM and trophectoderm outgrowths (S5 Table). On the other hand, dead embryos were *Eftud2* heterozygous (n = 1) or homozygous (n = 2) mutants (S5 Table). Our analysis further suggest that *Eftud2* homozygous mutant embryos did not hatch from the zona pellucida. Since failure to hatch *in vitro* is associated with abnormal blastocyst growth and survival [14], we postulate that the longer EFTUD2 protein is required for survival of pre-implantation embryos.

**Table 5:** Features of blastocysts cultured *in vitro* collected from matings of *Eftud2*^*+/-*^ with *Eftud2*^*+/-*^ mice in the CD1 genetic background.

Phenotype	Day 1	Day 2	Day 3	Day 4	Day 5
Compacted Morula	8	3	1	1	1
Blastocyst	5	4	1	0	0
Expanded blastocyst	30	10	5	3	2
Hatched	0	22	3	2	1
Trophectoderm and ICM outgrowth	0	0	24	27	29
Dead	1* (2.2)	4^#^(11.4)	5^‡^ (23.2)	1^$^ (25.0)	0 (25.0)
Healthy	43 (97.7)	39 (88.6)	34 (77.3)	33 (75.0)	33 (75.0)

N = 44, from 6 litters, (% of total)

*4-cell embryo

^#^1 compacted morula, 3 blastocysts (1 blastocyst was *Eftud2*^*+/-*^)

^‡^1 compacted morula, 4 blastocysts (1 blastocyst was *Eftud2* ^*-/-*^)

^$^1 blastocyst (*Eftud2*^*-/-*^)

## Discussion

In this study, we show that *Eftud2* was expressed throughout embryonic development, and more broadly than previously reported [13]. In addition, though a subset of MFDM patients have heart defects, *Eftud2* expression was only found in the developing heart at E9.5. Moreover, we report an essential role for *Eftud2* in mouse embryonic development, as early as E3.5, and demonstrate that reduction of EFTUD2 caused a developmental delay in heterozygous embryos in the two days that follow gastrulation. However, the delay was resolved by the onset of organogenesis, and heterozygous mice were born at the expected Mendelian frequency and do not model MFDM. This is the first report of a mouse model carrying a mutation in *Eftud2* in the literature.

The deletion of exon 2 of *Eftud2*, the first coding exon, is expected to remove 35 amino acids at the N-terminal of EFTUD2. Despite two additionnal methionine in exon 3 that could theoretically produce a functional truncated protein, our data demonstrate a reduction of EFTUD2 mRNA and protein. Thus we postulate that either the shorter transcript which lacks exon 2 is unstable or that it encodes for a truncated protein which is not stable. Interestingly, one of the predicted *EFTUD2* human transcripts does not contain exon 2 (S8 Fig). Although its role is currently unknown, our study suggests that the protein encoded by this transcript can not perform all of the same function as the longer EFTUD2 protein.

The N-terminal end of EFTUD2 has been studied in yeast and indicate that this portion of the protein is important in assembly and dissasembly of the U5-spliceosome, as well as in splicing. U5-spliceosome assembly/disassembly requires interaction of the N-terminal portion of Snu114, the yeast ortholog of EFTUD2, with Prp8[15], indicating that this region is important for interaction with other proteins in the U5 spliceosome. Interestingly, both the N and C termini of Snu114 are necessary for spliceosome activation [16], and deletion of the N-terminal end of Snu114 caused a temperature-sensitive lethality and splicing defects [17]. Our data demonstrating that heterozygous embryos with deletion of this exon have reduced EFTUD2 protein, suggest that this region may also be important for the stability of full-length EFTUD2 protein. Since the N-terminal region of the protein shows high sequence conservation between yeast, mouse (S9 Fig), and human [17], we propose that the function of this region of the protein may also be conserved. This is supported by our finding that deletion of the N-terminal domain results in early embryonic lethality of *Eftud2*^*-/-*^ mice.

In zebrafish models with perturbation of eftud2, neuronal apoptosis [11, 12] was associated with an increase in the p53 pathway [11]. This was rescued by mRNA injection of Upf1—a DNA/RNA helicase that plays a central regulatory role in NMD [18], suggesting that compromised NMD in eftud2^-/-^ was partly responsible for the upregulation in p53. In contrast, *Eftud2* heterozygous mutant mouse embryos had non-significant reduction in *P53* and *Upf1* mRNA (S2 Fig). Thus, the reduced level of EFTUD2 in heterozygous mouse embryos was sufficient for normal neuronal survival.

Our data demonstrate that EFTUD2 is required early in mouse embryogenesis, similar to other components of the spliceosome [19]. However, *Eftud2*^*-/-*^ embryos formed a blastocoel cavity, unlike embryos with knock-down of other components of the major spliceosome (*Sf3b14*, and *Sf3b)* which have disrupted formation of the blastocoel cavity and showed abnormal trophectoderm differentiation [19]. It is then possible that maternal deposition of core components of the spliceosome enables embryonic survival until the blastocyst stage. Additionally, although our data indicate that *Eftud2* homozygous mutant embryos die *ex vivo* because they are unable to hatch out of the zona pellucida, this is unlikely to be the case *in vivo* where enzymes in the uterus environment mediate hatching. Based on the roles described for *Eftud2* in zebrafish, we speculate that cell cycle arrest and upregulation of p53 is responsible for death of *Eftud2* homozygous mutant mice, *in vivo*. Whether or not this arrest is associated with intron retention, as was found in zebrafish will need to be further evaluated. Nonetheless, our studies indicate that EFTUD2 with the highly conserved N-terminus is required for survival post-implantation.

Although the timing of development pre-implantation differs between mouse and human, many molecular aspects of the process, including the expression of lineage-specific transcription factors, are similar [20]. Our findings, combined with the fact that *Eftud2* is expressed in early morulas and that homozygous mutations of *EFTUD2* has never been reported in humans, suggest that a certain threshold of EFTUD2 is necessary for early viability in both mice and humans. This threshold might differ between the two species at later stages of development. In humans, *EFTUD2* is haploinsufficient [4] whereas in our study, heterozygous animals on a mixed CD1;FvB genetic background were viable and fertile, without major molecular or phenotypic abnormalities. Although we observed a small reduction in the expected number of *Eftud2* heterozygous mice on a the C57BL/6 genetic background, we did not find craniofacial abnormalities in these mutants when they were examined before birth. In fact, we were unable to determine why these heterozygous animals died. Further analysis on the C57BL/6 are underway to determine the timing and cause of perinatal death. Interestingly, a number of human autosomal dominant syndromes are not recapitulated in heterozygous mouse models [21–23]. So far, the reasons for the discrepancy between mouse and human remains unknown. Since it is clear that the genetic background of the mouse model used is an important contributor [24, 25], analysis of mice with deletion of exon 2 in *Eftud2* on a different genetic background may yet reveal phenotypes associated with MFDM.

We report here, for the first time, that *Eftud2* mutation in mice causes early embryonic lethality. Hence, a conditional *Eftud2* knock-out mouse model is necessary to specifically study the requirement of this splicing factor during craniofacial development.

## Materials and methods

### Probe production

*In situ* probe for *Eftud2* was made using cDNA amplified from E10.5 CD1 wild-type embryos with DreamTaq (Invitrogen) using the following primers; Forward: gatgaattgattcggaatgtc Reverse: agaggtcagaatgtcctgttca. The PCR product of 908bp was cloned in the TOPO TA cloning kit (Invitrogen). The resulting constructs were analyzed by restriction analysis and Sanger sequencing. The plasmid was linearized with EcoRV and transcribed using Sp6 polymerase or linearized with Spe1 and transcribed with T7 polymerase to generate the sense and antisense probes, respectively. Transcription of probes was done using the DIG RNA Labeling Mix (Roche). All protocols were used according to the manufacturer’s instructions.

### Preparation of embryos for *in situ* hybridization and embedding

Wild-type CD1 dissected embryos were fixed in 4% paraformaldehyde overnight and dehydrated using a graded methanol series for wholemounts. Seven micrometer sections were performed on a Leica RM 2155 microtome and mounted on coated slide. Wholemount and sections *in situs* were performed as previously described [26]. Hematoxylin and Eosin (H&E) staining was performed using standard protocol.

### Generation of *Eftud2* mouse line using CRISPR/Cas9 gene-editing system

All procedures and experiments were performed according to the guidelines of the Canadian Council on Animal Care and approved by the Animal Care Committee of the Montreal Children’s Hospital. 4 single guide RNA (sgRNA) were designed flanking exon 2 of the mouse *Eftud2* gene using http://crispr.mit.edu/ website. sgRNA#1: ACCTTTCCTACCACGTAGGC; sgRNA#2: CCTTACCTTTCCTACCACGT; sgRNA#3: GAATGTTGTCTGTAACGGGA; sgRNA#4: GGAATGTTGTCTGTAACGGG. gRNA were transcribed *in vitro* using the GeneArt Precision gRNA Synthesis Kit (Thermofisher) following the manufacturer's instructions. The 4 gRNA were injected simultaneously with Cas9 mRNA (Sigma) in mouse zygotes from CD1-FVB mixed background or pure C57BL/6 background and transferred to a surrogate female for gestation. 2 mice from each background were recovered and mated to wild-type animals (CD1 or C57BL/6, respectively). Sanger sequencing of genomic DNA from their G1 offsprings confirmed deletion of exon 2 of *Eftud2* (S10A Fig).

To dilute any potential off-targets mutations introduced by CRISPR/Cas9 engineering, founders carrying the desired *Eftud2* mutation were backcrossed to wild-type CD1 or wild-type C57BL/6 mice. No phenotypic changes were found in embryos or pups analyzed 2–6 generations after backcrossing to wild-type mice. Therefore, these numbers have been pooled.

### Genotyping of *Eftud2*^*+/-*^ mice

Genomic DNA was extracted from mouse tails or yolk sacs as previously described [27]. Genotyping was performed using 3-primers targeting exon 2 of *Eftud2* using the following program: 30 sec 95°C, 30 sec 55°C, 30 sec 72°C for 35 cycles followed by an elongation step of 10 minutes at 72°C. As depicted in S10B and S10C Fig, the PCR condition was optimized to amplify wild-type (180bp) and mutant (265bp) amplicons. The following primers were used: *Eftud2* F1: atgaaccagggcagagaagt, *Eftud2* R1: tccaacagtagccaagccat, *Eftud2* R2: ccatgatgctaaaattcaaggag.

### Analysis of embryos from *Eftud2*^*+/-*^ mice matings

For embryo collection, the day of the presence of vaginal plug was considered embryonic day 0.5 (E0.5). Embryos were collected and yolk sacs were used for genomic DNA extraction for genotyping. For stage E3.5, blastocysts were flushed out of the uteri and single blastocyst was cultured in 96-well plate in DMEM with 10% FBS in a humidified incubator under 5% CO_2_ air atmosphere for up to 5 days. Morphology was followed daily under light microscopy. For stages E8.5 to E10.5, the number of somites were counted under light microscope (Leica MZ6 Infinity1 stereomicroscope) at time of dissection. Embryos were fixed in 4% paraformaldehyde at 4°C overnight, transferred in PBS and kept at 4°C.

### RNA isolation for RNA sequencing and RT-qPCR

E9.5 embryos on the mixed CD1;FVB genetic background were collected in RNA later and pooled as follow:

**wt1**: 2 embryos from litter#1 (19 and 23 somites)
**het1**: 2 embryos from litter#1 (23 and 24 somites)
**wt2**: 2 embryos from litter#2 (24 and 24 somites)
**het2**: 2 embryos from litter#2 (24 and 24 somites)
**wt3**: 2 embryos from litter#3 (24 and 24 somites)
**het3**: 2 embryos from litter#3 (23 and 25 somites)

RNA extraction was performed using Qiagen RNeasy kit following manufacturer's protocol. An aliquot was sent for RNA sequencing analysis. Total RNA was treated with DNAse (NEB, according to manufacturer's protocol) and used for reverse transcription with the iScript cDNA synthesis kit (Bio-rad, Cat. #170–8890, according to the manufacturer's protocol). qRT-PCR was performed using the ssoAdvanced universal SYBR green supermix (Bio-Rad, cat#172–5270) on a Roche LightCycle 480 PCR machine. qPCR experiments were performed in duplicates to ensure technical replicability. Target genes were normalized with the normalization factor as calculated by geNorm software (v3.4; Ghent university hospital center for medical genetics)[28]. Three house-keeping genes including B2M, GAPDH, and SDHA were used for generation of the normalization factor as previously reported [28]. RT-PCR program included a hot start at 95°C for 5 min, followed by 40 cycles of a denaturation step at 95°C for 10s and an annealing/extension step at 60°C for 30s. Primers used were the following:

*Eftud2* forward (exon2): GCACTCGGCTGAGCATTC
*Eftud2* reverse (exon2): ATCCTCGTCCTCGTCCTCAT
*Eftud2* forward (exon15-16): GATCGAGCATACCTACACTGGC
*Eftud2* reverse (exon15-16): GTACATCTTCGTCGTGTGGCA
*Casp8* forward: ggcctccatctatgacctga
*Casp8* reverse: tgtggttctgttgctcgaag
*Bbc3* forward: tgtgaccactggcattcatt
*Bbc3* reverse: cccagactcctccctcttct
*Trp53* forward: gcttctccgaagactggatg
*Trp53* reverse: gtccatgcagtgaggtgatg
*Upf1* forward: AGCTCGACGCACAAGTTGG
*Upf1* reverse: CGCAGGCAGGATCATGGATT
*Sdha* forward: GCTGTGGCCCTGAGAAAGATC
*Sdha* reverse: ATCATGGCCGTCTCTGAAATTC
*B2M* forward: ATGCTATCCAGAAAACCCCTCAA
*B2M* reverse: GCGGGTGGAACTGTGTTACG
*Gapdh* forward: ATGACATCAAGAAGGTCCTG
*Gapdh* reverse: CATACCAGGAAATGAGCTTG

### RNA-sequencing analysis

Sequencing libraries were prepared by Genome Quebec Innovation Centre (Montreal, Canada), using the TruSeq Stranded Total RNA Sample Preparation Kit (Illumina TS-122-2301, San Diego, California, United States) by depleting ribosomal and fragmented RNA, synthesizing first and second strand cDNA, adenylating the 3′ ends and ligating adaptors, and enriching the adaptor-containing cDNA strands by PCR. The libraries were sequenced using the Illumina HiSeq 4000 PE100 sequencer, 100 nucleotide paired-end reads, generating approximately 60 million reads sample. The sequencing reads were trimmed using CutAdapt [29] and mapped to the mouse reference genome (mm10) using STAR [30] aligner (version 2.4.0e), with default parameters, and annotated using the Gencode [31] M2 (version M2, 2013) annotation. htseq-count (part of the ‘HTSeq’ [32] framework, version 0.5.4p5.) was used for expression quantification.

We performed a principal component analysis (PCA) on all genes and a hierarchical clustering on the 1000 most variable genes, and used DESeq [33] for differential expression analysis. A gene set list of interest was derived by applying filters on gene expression, such that only those with p-adj < 0.05 and FoldChange > = 1.5 and p-adj < 0.05 and FoldChange < = 0.67 were selected as up- and down-regulated genes.

To perform a differential splicing analysis, we first selected expressed genes in heterozygous and wild-type embryos using the total number of mapping reads (gene count). We chose genes with a gene count-mean greater than or equal to 100 in both groups. Then, we picked junctions on those genes, by retaining junctions with mean count greater than or equal to 10 in at least one group of embryos.

In order to identify differential splicing events across samples, we used a splice-junction based approach. An event of interest is represented by a set of junctions that either share a common start, or conversely share a common end. For each junction in each set, a usage percentage was computed. A t-test was performed on the usage percentage of each junction. Junctions that have a p-value less than 0.05 and an absolute difference of usage percentage greater than or equal to 0.20 between the two groups were considered significant.

To determine if there is a difference in the percentage of unannotated junctions between wild-type and heterozygous samples, we first selected expressed genes in both groups of samplesand, for each sample, junctions overlapping those genes. Annotated junctions were defined using Gencode M2 annotation. The percentage of unannotated junctions was computed for each sample and a t-test was performed in order to compare the two groups.

To estimate the presence of *Eftud2* exon 2 in *Eftud2* transcripts, the inclusion level of *Eftud2* exon2 was determined for each sample, using the inclusion (I) and skipping (S) junction counts, as follow: Ψ = (I/2)/((I/2)+S). Then, we computed the mean and the standard deviation of Ψ for each group.

### Western Blot analysis

Snap-frozen E11.5 embryos on the mixed CD1;FVB genetic background were lysed in RIPA buffer (25 mM Tris∙HCl pH 7.6, 10% glycerol, 420 mM NaCl, 2 mM MgCl_2_, 0.5% NP-40, 0.5% Triton X-100, 1 mM EDTA, protease inhibitor) on ice. Embryos were sonicated and centrifuged at 13000rpm for 20 minutes at 4°C. Clarified protein lysates were measured according to standard methods using a DC protein assay kit (Bio-Rad, Mississauga, Ontario, Canada). 50μg of protein was resolved on 10% TGX Stain-Free gels (Bio-Rad, Cat#4568045) and activated by exposure to UV light for 1 min to visualize total proteins. They were then transferred to low fluorescence PVDF membranes (Bio-Rad, Cat#1620260), and a stain-free blot image was acquired to obtain a total protein profile, as previously described [27]. After blocking in 5% milk, all membranes were probed with primary antibody (EFTUD2 1:1000, Sigma, cat#SAB2701211). Immunoblotted proteins were visualized using horseradish peroxidase-conjugated secondary antibody (Cell Signaling), and antigen-antibody complexes were detected using the ECL system (ZmTech Scientifique, Montreal, Quebec, Canada). Images of western blots were taken with Bio-Rad’s ChemiDoc MP System and were digitally analyzed using Image Lab software. The total protein profile was used as a loading control to normalize the level of the protein of interest.

### Statistical analysis

Two-tailed unpaired t-test analysis was calculated using Excel and Prism Software and Chi-square test was calculated using Prism. Significant p-values are represented as *P<0.05, **P<0.01 and ***P<0.001.

## Supporting information

S1 Fig ISH experiments showing results obtained with both sense and antisense probes. Representative images of WMISH and tissue sections *in situ* hybridization of E8.5 (A) E9.5 (B) and E10.5 (C) CD1 embryos are shown.
(EPS)

S2 Fig Genes involved in the p53 or NMD pathways are not perturbed in *Eftud2* heterozygous embryos. mRNA levels of genes involved in the p53 pathways namely (A) *caspase-8*, *Bcb3* and *p53*, as well as (B) *Upf1*, involved in NMD, were analyzed in E9.5 embryos by qRT-PCR. WT = 3, HET = 3. Results represent average ± SEM.
(EPS)

S3 Fig PCA of heterozygous and wild-type embryos. PCA analysis of gene expression in wild-type and heterozygous E9.5 embryos. These samples are represented respectively in black and white. Each sample is a pool of 2 somite-matched embryos (see Materials and Methods for samples description). Samples grouped based on their litter instead of their genotype.
(EPS)

S4 Fig Hierarchical clustering of gene expression in heterozygous and wild-type embryos. Hierarchical clustering was performed on wild-type and heterozygous E9.5 embryos. Three clusters are observed consisting of a heterozygous sample and a wild-type samples collected from the same litter.
(EPS)

S5 Fig Inclusion levels of *Eftud2* exon2. *Eftud2* exon2 is present in 100% of transcripts in wild-type embryos (WT) while present in 50% of transcripts in heterozygous embryos (HET).
(EPS)

S6 Fig *Eftud2* heterozygous embryos are similar to wild-type just prior to birth. E18.5 whole embryos were (A) weighted and (B) measured. Associated placentas were also weighted (C). Ratio between embryos and placental weights (D). WT = 15; HET = 23.
(EPS)

S7 Fig Representation of the features analyzed of E3.5 embryos grown *ex vivo*. ICM: inner cell mass.
(EPS)

S8 Fig Line bar of 5’ end of *Eftud2* gene. Human *EFTUD2* transcript NM 001142605 does not contain exon 2. Exon 3 in both species contains several methionines.
(EPS)

S9 Fig Protein sequence alignment of mouse Eftud2 and human EFTUD2. Underlined sequenced is the region encoded by exon 2. Variation in amino acids between the two sequences is shown is red.
(EPS)

S10 Fig Genotyping of mouse with *Eftud2* exon 2 deletion. (A) Sequence representing deletion of exon 2 (in grey) flanked by cutting sites of the 2 guide RNAs used to generate this mutant alelle (in red). Two additional guide RNAs used for the microinjection (in blue) are also shown. (B) Genotyping strategy to identify *Eftud2* exon 2 deletion using a 3 primers PCR (red arrows). (C) Gel representing PCR products obtained from tails genomic DNA. PCR conditions were optimized to obtained a wild-type band of 180bp and a mutant band of 265bp.
(EPS)

S1 Table List of differentially expressed genes in heterozygous embryos compared to the wild-type. (DOCX)

S2 Table List of differentially used junctions. (DOCX)

S3 Table Percentage of unannotated junctions per sample. (DOCX)

S4 Table Features of blastocysts cultured *in vitro* collected from matings of *wild-type* male and female mice on the mixed CD1;FvB genetic background. (DOCX)

S5 Table Genotypes of E3.5 embryos after 5 days of culture *in vitro* collected from matings of *Eftud2*^*+/-*^ with *Eftud2*^*+/-*^ mice in the CD1 genetic background. (DOCX)
