# Supplementary material for: Loss of function mutation of Eftud2, the gene responsible for mandibulofacial dysostosis with microcephaly (MFDM), leads to pre-implantation arrest in mouse
Source: PLoS One. 2019 Jul 5;14(7):e0219280. doi: 10.1371/journal.pone.0219280 (PMC6611600; doi:10.1371/journal.pone.0219280)
Supplement: S1 Table — (DOCX) [file pone.0219280.s011.docx]

**S1 Table. List of differentially expressed genes in heterozygous embryos compared to the wild-type.**

| **Genes Symbol** | **Genes Name** | **Fold change** | **Adj P value** |
| --- | --- | --- | --- |
| 5830417I10Rik | RIKEN cDNA 5830417I10 gene | 2.332 | 1.14E-15 |
| Nr1h5 | Nuclear receptor subfamily 1, group H, member 5 | 9.702 | 1.22E-05 |
| Pnpo | Pyridoxamine 5'-Phosphate Oxidase | 1.525 | 0.000210723 |
| Gm9816 | Predicted pseudogene 9816 | 3.696 | 0.002063388 |
