# Supplementary material for: Loss of function mutation of Eftud2, the gene responsible for mandibulofacial dysostosis with microcephaly (MFDM), leads to pre-implantation arrest in mouse
Source: PLoS One. 2019 Jul 5;14(7):e0219280. doi: 10.1371/journal.pone.0219280 (PMC6611600; doi:10.1371/journal.pone.0219280)
Supplement: S2 Table — (DOCX) [file pone.0219280.s012.docx]

**S2 Table. List of differentially used junctions.**

| **Main junction** | **Alternative junction** | **Genes Symbol** | **Junction usage difference** | **P value** | **Adjusted p-value** |
| --- | --- | --- | --- | --- | --- |
| chr11:102871186-102877609 | chr11:102871186-102880894 | Eftud2 | 0.753 | 0.001 | 0.902 |
| chr11:102877719-102880894 | chr11:102871186-102880894 | Eftud2 | 0.420 | 0.029 | 0.902 |
| chr11:120725897-120726087 | chr11:120726007-120726087 | Dcxr | 0.275 | 0.020 | 0.902 |
| chr11:71019724-71020005 | chr11:71019724-71024549 | Mis12 | 0.253 | 0.035 | 0.902 |
| chr11:96293204-96293374 | chr11:96291266-96293374 | 0610040B09Rik | 0.246 | 0.025 | 0.902 |
| chr8:12863863-12864919 | chr8:12861725-12864919 | Atp11a | 0.242 | 0.004 | 0.902 |
| chr7:48880402-48880660 | chr7:48880402-48881407 | E2f8 | 0.225 | 0.033 | 0.902 |
| chr12:77277456-77323844 | chr12:77277456-77331895 | Fut8 | 0.219 | 0.022 | 0.902 |
| chr1:57967724-57969406 | chr1:57967724-57970020 | Kctd18 | 0.214 | 0.015 | 0.902 |
| chr7:98637521-98641492 | chr7:98637521-98639730 | 2210018M11Rik | 0.212 | 0.005 | 0.902 |

Junctions that have a common start or end are grouped together as first and alternative junctions. The main junction corresponds to the junction that is more present in wild-type samples.
