# Supplementary material for: Loss of function mutation of Eftud2, the gene responsible for mandibulofacial dysostosis with microcephaly (MFDM), leads to pre-implantation arrest in mouse
Source: PLoS One. 2019 Jul 5;14(7):e0219280. doi: 10.1371/journal.pone.0219280 (PMC6611600; doi:10.1371/journal.pone.0219280)
Supplement: S3 Table — (DOCX) [file pone.0219280.s013.docx]

**S3 Table. Percentage of unannotated junctions per sample**

| **sample** | **wt1** | **wt2** | **wt3** | **het1** | **het2** | **het3** | **P value** |
| --- | --- | --- | --- | --- | --- | --- | --- |
| **percentage of unannotated junctions** | 0.023 | 0.021 | 0.020 | 0.023 | 0.021 | 0.019 | 0.833 |
