# Supplementary material for: Loss of function mutation of Eftud2, the gene responsible for mandibulofacial dysostosis with microcephaly (MFDM), leads to pre-implantation arrest in mouse
Source: PLoS One. 2019 Jul 5;14(7):e0219280. doi: 10.1371/journal.pone.0219280 (PMC6611600; doi:10.1371/journal.pone.0219280)
Supplement: S4 Table — (DOCX) [file pone.0219280.s014.docx]

**S4 Table. Features of blastocysts cultured *in vitro* collected from matings of *wild-type* male and female mice on the mixed CD1;FvB genetic background.**

| **Phenotype** | **Day 1** | **Day 2** | **Day 3** | **Day 4** | **Day 5** |
| --- | --- | --- | --- | --- | --- |
| Compacted morula | 0 | 0 | 0 | 0 | 0 |
| Cavitated | 0 | 0 | 0 | 0 | 0 |
| Blastocyst | 21 | 15 | 9 | 7 | 3 |
| Hatched | 4 | 7 | 2 | 2 | 4 |
| Trophectoderm and ICM outgrowth | 0 | 3 | 14 | 16 | 18 |
| Dying | 0 | 0 | 0 | 0 | 0 |
| Healthy embryos | 25 | 25 | 25 | 25 | 25 |

N=25 blastocysts from 2 litters
