# Supplementary material for: Loss of function mutation of Eftud2, the gene responsible for mandibulofacial dysostosis with microcephaly (MFDM), leads to pre-implantation arrest in mouse
Source: PLoS One. 2019 Jul 5;14(7):e0219280. doi: 10.1371/journal.pone.0219280 (PMC6611600; doi:10.1371/journal.pone.0219280)
Supplement: S5 Table — (DOCX) [file pone.0219280.s015.docx]

**S5 Table. Genotypes of E3.5 embryos after 5 days of culture *in vitro* collected from matings of *Eftud2^+/-^* with *Eftud2^+/-^* mice in the CD1 genetic background.**

| **Genotype** | **Trophectoderm and ICM outgrowth** | **Dead** | **TOTAL** |
| --- | --- | --- | --- |
| *Eftud2 +/+* | 4 | 0 | 4 |
| *Eftud2 +/-* | 9 | 1 | 10 |
| *Eftud2 -/-* | 0 | 2 | 2 |

N=16 from 2 litters
